# Individual and institutional factors influencing dentists’ practice in underserved areas

**DOI:** 10.1038/s41598-025-32094-8

**Published:** 2026-01-05

**Authors:** Hawazin W. Elani, Ningsheng Zhao, Helena S. Schuch, Greg Saldutte, Elizabeth Mertz, Marko Vujicic

**Affiliations:** 1 Department of Oral Health Policy and Epidemiology, Harvard School of Dental Medicine, 188 Longwood Avenue, Boston, MA 02115 USA; 2 Department of Health Policy and Management, Harvard T. H. Chan School of Public Health, Boston, MA USA; 3 School of Dentistry, The University of Queensland, Brisbane, QLD Australia; 4 Health Policy Institute, American Dental Association, Chicago, IL USA; 5 Department of Preventive and Restorative Dental Sciences, School of Dentistry, University of California, San Francisco, USA

**Keywords:** Dental workforce distribution, Underserved areas, Explainable machine learning, SHAP values, Demographic factors, Institutional characteristics, Health care, Medical research

## Abstract

Access to dental care is a key determinant of oral health, yet disparities in provider distribution across the United States contribute to inequitable access, particularly in underserved areas. To predict dentists’ likelihood of practicing in underserved settings and identify factors influencing practice location decisions, we developed explainable machine learning models using national data from 56,175 dentists who graduated between 2000 and 2022. We examined 76 predictors including individual- and dental school-level characteristics and defined outcomes as practicing in Federally Qualified Health Centers, dental shortage areas, or rural dental shortage areas. Our models demonstrated strong predictive performance, and the results revealed that general practice specialty, fewer years of experience, non-owner practice status, and demographic factors such as gender and race were strongly associated with practicing in underserved areas. Institutional characteristics, including dental school location and diversity index, also played a significant role. The relationship between educational debt, experience, and practice outcomes varied by practice type and race/ethnicity. These findings highlight the value of explainable machine learning in informing targeted workforce policies that address individual, institutional, and demographic drivers of dentist distribution to improve access to dental care in underserved communities.

**Supplementary Information:**

The online version contains supplementary material available at 10.1038/s41598-025-32094-8.

## Introduction

 Access to dental care is a key determinant of oral health, yet significant disparities in the geographic distribution of dental providers persist throughout the United States. National projections suggest that the aggregate supply of dentists may meet the demand by 2030, assuming current utilization trends continue​.^1^ However, these estimates obscure major regional and community-level imbalances. The US Health Resources and Services Administration (HRSA) projects a 9% growth in the dental workforce from 2017 to 2030, a rate approximately equivalent to population growth. Despite this projected growth, about 24.7 million individuals currently live in dental care shortage areas,^2^ with rural communities disproportionately impacted. Rural regions experience severe shortages, with approximately one dentist per 3,850 residents, compared to one per 1,470 in urban areas. Furthermore, 387 counties nationwide experience pronounced disparities in dental clinic availability, underscoring the uneven distribution of oral healthcare providers^2^.

The maldistribution of dentists mirrors broader systemic workforce challenges within the US healthcare landscape. The Association of American Medical Colleges projects a national physician shortfall ranging from 13,500 to 86,000 by 2036, reinforcing concerns about the geographic distribution of healthcare professionals more broadly^3^. Dental workforce shortages similarly affect underserved populations, particularly those in rural areas and low-income communities. Federally Qualified Health Centers (FQHCs), a key safety-net provider, experience persistent staffing shortages, including dental professionals, limiting access to essential dental services and worsening oral health disparities^4^.

Contributing factors to this maldistribution include high levels of dental school debt,^5^ an aging workforce,^6^ geographic isolation, and inadequate reimbursement for providers serving low-income populations^4^. Recognizing these challenges, federal and state initiatives have introduced loan repayment programs and HRSA-funded recruitment initiatives designed to attract and retain dental providers, particularly those from underrepresented minority backgrounds, in high-need areas^4^. However, the long-term effectiveness of these policy interventions and the extent to which dental education programs promote service in underserved regions is still inadequately understood^4,7^.

Therefore, understanding the determinants that influence dentists’ decisions regarding practice location is essential for developing effective workforce policies. Traditional interpretable methods such as logistic regression have been widely used to examine these determinants. However, such models often assume linear and additive relationships, which may not fully capture the complex interactions among those demographic, educational, and geographical factors that shape practice location decisions. Recent advances in machine learning (ML) allow for modeling these nonlinearities and high-dimensional interactions, potentially improving prediction accuracy and revealing nuanced patterns in provider behavior.

At the same time, the application of ML in health workforce research has been limited by concerns about interpretability. To address this gap, we use explainable artificial intelligence (XAI) techniques that combine the predictive strength of ML with transparent interpretation tools. These methods enable us to improve prediction of dentists’ likelihood of practicing in underserved areas and also to identify and visualize how key features such as educational background, training setting, and local context contribute to those predictions.

**Fig. 1:**
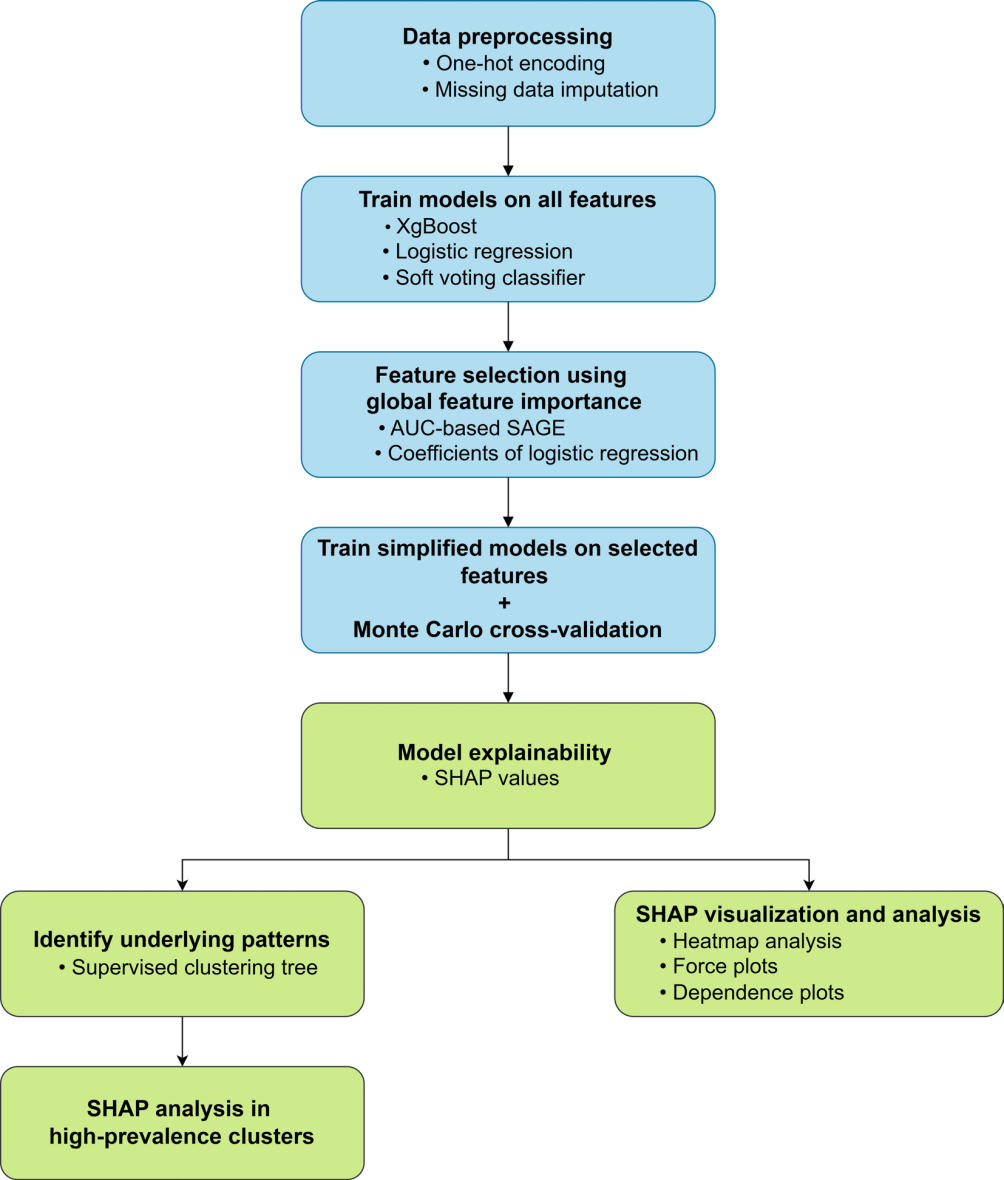
Flowchart of the data processing and explainable machine learning pipeline. *Note*. The proposed pipeline integrates supervised learning methods (e.g., soft voting classifier) for prediction, unsupervised learning techniques (e.g., proposed clustering tree) to identify underlying patterns, global model explainability approaches (e.g., AUC-based SAGE) for feature importance assessment, and local model explainability methods (e.g., SHAP) for interpreting individual predictions.

Accordingly, this study aims to develop and evaluate a set of ML models to predict dentists’ likelihood of practicing in underserved areas, including FQHCs, dental shortage areas, and rural dental shortage regions. We further apply XAI techniques to identify complex dependencies that may be overlooked by conventional regression models, even when those models demonstrate strong performance. Our explainable ML pipeline (Fig. 1) integrates both supervised and unsupervised learning with global and local model explainability to examine key factors that shape dentists’ workforce distribution. As demographic, economic, and policy landscapes evolve, identifying dentists who are more likely to serve in high-need communities can inform policymakers and dental education programs in creating targeted recruitment and retention strategies to improve access to oral healthcare for vulnerable populations.

## Methods

### Data sources and study samples

We constructed a dataset of dentist practice patterns in the US by combining data from multiple sources (Supplemental Fig. 1). We used data from the Survey of Dental Graduates (SDG) from 2000 to 2022, administered annually by the American Dental Association’s (ADA) Heath Policy Institute, which survey recent graduates from all Commission on Dental Accreditation (CODA)-accredited programs^8^. We also included data from the Survey of Dental Education (SDE) from 2000 to 2022, an annual CODA survey that includes detailed institutional data on dental schools, including enrollment numbers, tuition, and curriculum structures^9^. Lastly, we used a November 2022 snapshot from the ADA Office Database (ODB), a comprehensive census of all active US dentists, including practice location, type, and specialty^10^.

### Outcome measures

We examined three outcomes related to underserved dental practice settings. First, practice in general dental shortage areas, defined as regions with fewer than 1 dentist per 5000 population^2,11^. Second, the likelihood of practicing in rural dental shortage areas, defined using the National Center for Health Statistics (NCHS) Urban-Rural Classification Scheme, which includes dental shortage areas in micropolitan and noncore counties^12^. Third, practice within FQHCs, based on dentists’ self-reported practice settings.

### Predictor variables

The final analytic dataset included 76 predictors categorized into individual-level and school-level characteristics. Individual-level variables included demographics (age, race and ethnicity), years of experience, and specialty. School-level variables included measure of racial and ethnic diversity “diversity indices”, class size, geographic location, and tuition (full list of variables in Supplemental Table 1). Variables with high missingness (> 60%) or strong collinearity (correlation > 0.8) were excluded from the analysis. In secondary analyses, we examined individual-level and school-level predictors independently to evaluate their contribution to practice location decisions.

### Model development and evaluation

Prior to modeling, we applied data preprocessing techniques. Missing data were imputed using median values, categorical variables were converted into binary indicators (“one-hot encoding”), and continuous variables were standardized (z-scores normalization) to ensure comparability across features.

We developed predictive models using three classification algorithms. First, we used the eXtreme Gradient Boosting (XGBoost) classifier, a gradient-boosting framework, for its ability to handle nonlinear relationships and complex interactions among predictors^13^. The XGBoost predictions were calibrated using isotonic calibration to enhance probabilistic accuracy^14^. Second, logistic regression served as a baseline model for interpretability and capture linear relationships between predictors and outcomes. Finally, we combined these models into a soft voting classifier ensemble to enhance predictive robustness and generalizability^15^. Separate models were developed for each outcome independently.

The models were initially tuned on a randomly selected validation set. To ensure robustness and stability, their performance was subsequently assessed using Monte Carlo cross-validation with random 80/20 splits across 30 iterations^16^. Because relatively few dentists practiced in underserved areas, the outcomes were highly imbalanced, with the positive class (practice in underserved settings) representing a small share of total observations. This imbalance can bias standard accuracy metrics toward the majority class, potentially overstating performance. Therefore, we used performance metrics that are more informative under class imbalance, including the Area Under the Receiver Operating Characteristic Curve (AUC), sensitivity, specificity, precision, the lift score, and the Matthews Correlation Coefficient (MCC)^17^. The classification-threshold, the cutoff used to convert predicted probabilities into binary class labels, was selected by identifying the value that maximized the MCC on the training data.

### Feature selection and importance ranking

To identify the most influential predictors, we used the Shapley Additive Global Importance (SAGE) method, an explainability approach that quantifies how much each variable contributes to model performance^18^. We modified the standard SAGE implementation to use the AUC as the performance metric to better handle class imbalance. Additionally, given the strong performance of logistic regression, we also used its coefficients as a global feature importance method. Features were selected separately for each outcome to maintain optimal model specificity and interpretability. To assess the impact of feature inclusion, we constructed inclusion curves showing performance changes as predictors were incrementally added according to their ranked importance^18^.

### Model interpretability and explainability

To improve model transparency and interpretability, we used SHapley Additive Explanations (SHAP) to analyze local feature importance to each prediction^19^. For the XGBoost model, we applied TreeSHAP for efficient feature attribution within decision trees^20^. We used Linear SHAP for logistic regression models to clarify model coefficient impacts^19^. For the soft voting classifier, we implemented Marginal SHAP to assess feature contributions in a model-agnostic manner^21^.

### SHAP values visualization

We used the SHAP dependence plots to assess the relationships between years of experience, debt after graduation, and practice outcomes to explore nonlinearities and interactions between these predictors and dentists’ likelihood of practicing in underserved areas. Additionally, we generated SHAP heatmaps to visualize predictors’ contribution across sorted instances for each outcome. SHAP force plots were also utilized to highlight how specific combinations of predictors influenced dentists’ decisions to practice in underserved areas.

### Supervised clustering and dimensionality reduction

To identify latent patterns within workforce distribution data, we applied the supervised clustering approach based on SHAP values^22^. Unlike traditional clustering, which groups data based on raw features, this method leverages model-informed explanations to form clusters that align more closely with the predictive structure of the data. As a result, the identified clusters are better separated and more meaningful in the context of the outcome of interest.

Furthermore, to improve clustering performance in low-dimensional spaces while effectively capturing underlying patterns, we propose a novel supervised clustering tree algorithm. As demonstrated in Supplemental Fig. 2, at each split in the tree, we first used Principal Component Analysis (PCA) to reduce the dimensionality of the SHAP values within the parent cluster. The resulting components were then subject to clustering methods to further partition the data. This hierarchical approach enhances interpretability by grouping dentists into three distinct types of clusters-high, average, and low prevalence- based on their likelihood of practicing in underserved areas. Within high-prevalence clusters, we analyzed the distribution of SHAP values to identify the key factors influencing dentists’ decisions to serve these communities. To evaluate clustering performance at each node, we used the Silhouette Score to ensure that the partitions were both distinct and well-formed^23^.

## Results

Our sample included 56,175 dentists, of whom 2,591 (4.6%) worked in FQHCs, 679 (1.2%) in dental shortage areas, and 405 (0.7%) in rural dental shortage areas (Table 1), indicating significant class imbalance. This imbalance reflects the actual shortage of dental providers in underserved areas. The sample was predominantly male (55.2%), with an average age of 41.2 years and an average experience of 12.3 years. Most respondents identified as White (66.1%). At the school level, the average diversity index was 0.53, and the average proportion of US citizens was 92.6%. Debt after graduation varied, with the highest average among dentists practicing in rural shortage areas ($266,825).

**Table 1:** Description of the study sample.

	Full sample	Federally qualified health centers		Dental Shortage area	Rural dental shortage area	
Characteristic	*N* = 56,175	*n* = 2,591		*n* = 679	*n* = 405	
Individual-Level, *n* (%)						
*Sex*						
Male	31,007 (55.2)	1,112 (42.9)		416 (61.3)	249 (61.5)	
Female	25,168 (44.8)	1,479 (57.1)		263 (38.7)	156 (38.5)	
Age, mean (SD)	41.2 (6.7)	39.7 (6.6)		40.5 (6.6)	40.3 (6.6)	
Experience, mean (SD)	12.3 (5.6)	10.6 (5.2)		11.7 (5.4)	11.4 (5.2)	
Race and ethnicity						
Asian	10,632 (18.9)	466 (18)		47 (6.9)	22 (5.4)	
Black	1,879 (3.3)	201 (7.8)		27 (4.0)	15 (3.7)	
Hispanic	1,818 (3.2)	118 (4.6)		20 (2.9)	11 (2.7)	
White	37,118 (66.1)	1545 (59.6)		523 (77.0)	319 (78.8)	
Other	4,728 (8.5)	261 (10.0)		62 (9.1)	38 (9.4)	
Specialty						
General Practice	43,928 (78.2)	2319 (89.5)		635 (93.5)	383 (94.6)	
Oral Surgery	1855 (3.3)	19 (0.7)		2 (0.3)	1 (0.2)	
Endodontics	1660 (3.0)	21 (0.8)		0 (0.0)	0 (0.0)	
Orthodontics	3216 (5.7)	30 (1.2)		19 (2.8)	10 (2.5)	
Pediatric dentistry	3402 (6.1)	141 (5.4)		17 (2.5)	8 (2.0)	
Periodontics	1136 (2.0)	19 (0.7)		1 (0.1)	0 (0.0)	
Other	978 (1.7)	42 (1.6)		5 (0.7)	3 (0.7)	
Debt after graduation, mean $ (SD)	243,968 (180,254)	269,171 (163,271)		255,370 (176,724)	266,825 (192,149)	
*School-Level, mean (SD)*						
Diversity index	0.53 (0.15)	0.54 (0.15)		0.46 (0.15)	0.45 (0.15)	
School size	423 (228)	428 (225)		353 (160)	364 (177)	
Proportion of Male students	53.9 (7.2)	53 (6.8)		55.5 (7.1)	55.7 (6.7)	
Proportion of US citizens	92.6 (8.5)	92.9 (7.8)		95.9 (6.1)	95.9 (5.6)	
Proportion of local state students	62.6 (33.4)	58.6 (25.8)		69 (24.1)	67.7 (23.4)	
GPA/DAT ratio	0.17 (0.01)	0.17 (0.01)		0.17 (0.01)	0.17 (0.01)	
Number of applications	1146 (1151)	1177 (1143)		760 (851)	776 (830)	
PhD program offered	24,478 (43.6)	1,113 (43)		352 (51.8)	210 (51.9)	

Note. Author analysis of data from the Survey of Dental Graduates (2000–2022), the ADA Office Database (2022), and the Survey of Dental Education (2000–2022).

### Model performance

After one-hot encoding, the data expanded to high-dimensional feature space with over 250 variables. However, the inclusion curve (Supplemental Fig. 3) indicated that more than half of these features were redundant or negatively impacted model performance. To improve model simplicity and predictive power, we retained the top 110, 60, and 50 features for predicting FQHCs, general dental shortage areas, and rural dental shortage areas, respectively.

Our models demonstrated strong performance in predicting dentists practicing in underserved settings. For FQHC, the best AUC was 0.82 (95% CI: 0.81–0.85) with an average AUC of 0.80 (95% CI: 0.80–0.81), sensitivity of 0.45, specificity of 0.93, precision of 0.23 (with a lift of 5.05), and MCC of 0.28 (Table 2). For general dental shortage areas, the best AUC was 0.83 (95% CI: 0.79–0.87) with average AUC of 0.80 (95% CI: 0.79–0.81), sensitivity of 0.70, specificity of 0.81, precision of 0.04 (with a lift of 3.58), and MCC of 0.14. For rural dental shortage areas, the best AUC was 0.84 (95% CI: 0.78–0.89), an average AUC of 0.80 (95% CI: 0.79–0.81), sensitivity of 0.82, specificity of 0.74, precision of 0.02 (with a lift of 3.07), and MCC of 0.11.

**Table 2:** Model performance in predicting dentists’ likelihood of practicing in underserved areas.

Metrics	Federally qualified health centers	Dental Shortage Area	Rural Dental Shortage Area
*Best Split*			
AUC (95% CI)	0.82 (0.81, 0.85)	0.83 (0.79, 0.87)	0.84 (0.78, 0.89)
Sensitivity	0.45	0.70	0.82
Specificity	0.93	0.81	0.74
Precision	0.23	0.04	0.02
Lift	5.05	3.58	3.07
MCC	0.28	0.14	0.11
*30 Splits*			
Average AUC	0.80 (0.80, 0.81)	0.80 (0.79, 0.81)	0.80 (0.79, 0.81)

Note. AUC, area under the receiver operating characteristic curve. The performance of these models was assessed using Monte-Carlo cross-validation, where the dataset was randomly split into 80% training and 20% testing over 30 iterations. The ‘Best Split’ represents the highest-performing single iteration, while ‘30 Splits’ refers to the average performance across all 30 iterations, providing a more stable estimate of model performance.

### SHAP analysis of key predictors

**Fig. 2:**
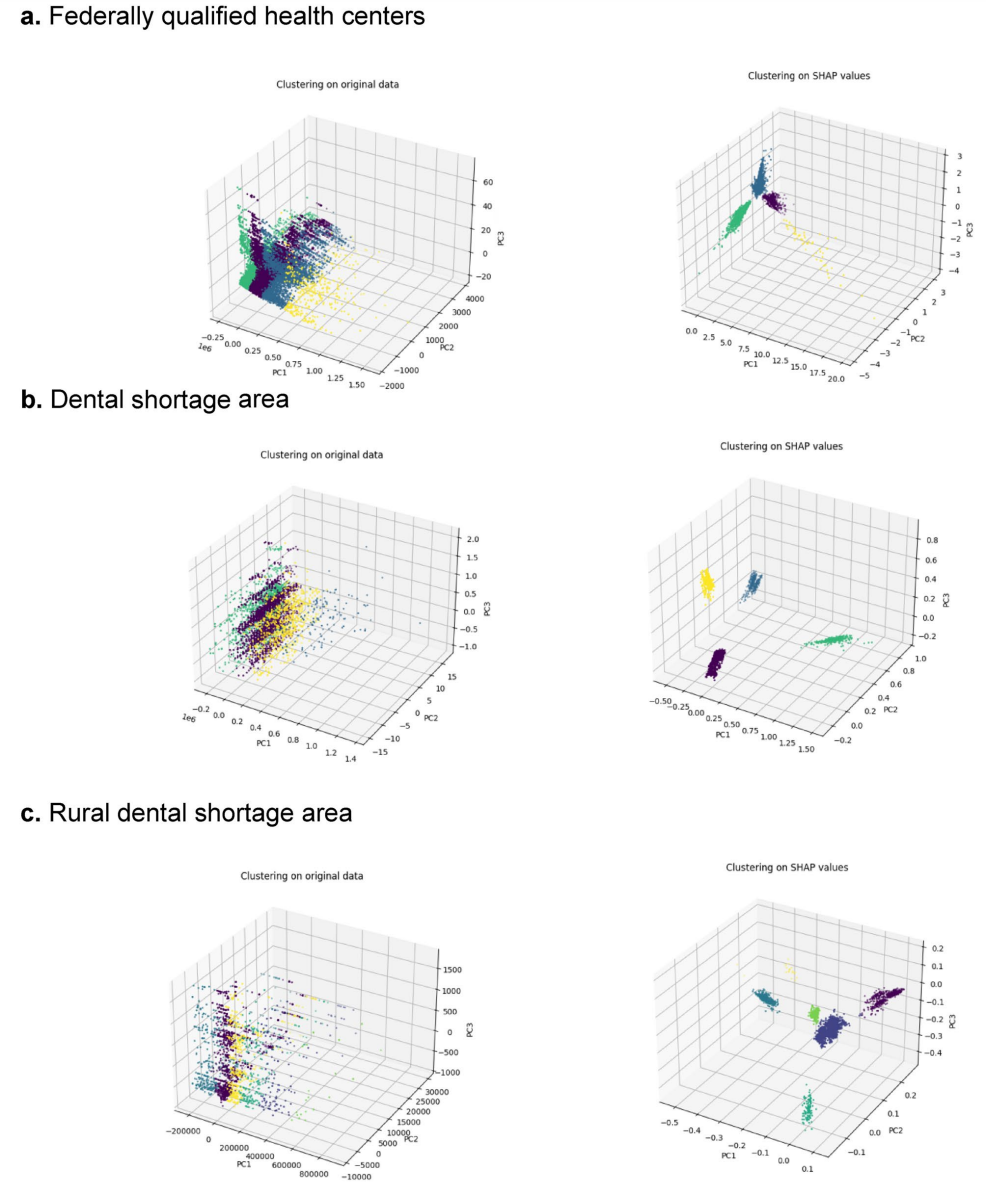
Examples of 3D plots from supervised clustering tree branches *Notes*. Unlike traditional clustering on the original data, supervised clustering tree results in better-separated and more meaningful clusters aligned with the model’s predictive structure.

The supervised clustering tree algorithm identified meaningful patterns, resulting in well-separated clusters, with Silhouette Scores exceeding 0.4 at nearly all branches. Figure 2 presents examples of 3D plots illustrating supervised clustering at selected branches.

**Fig. 3:**
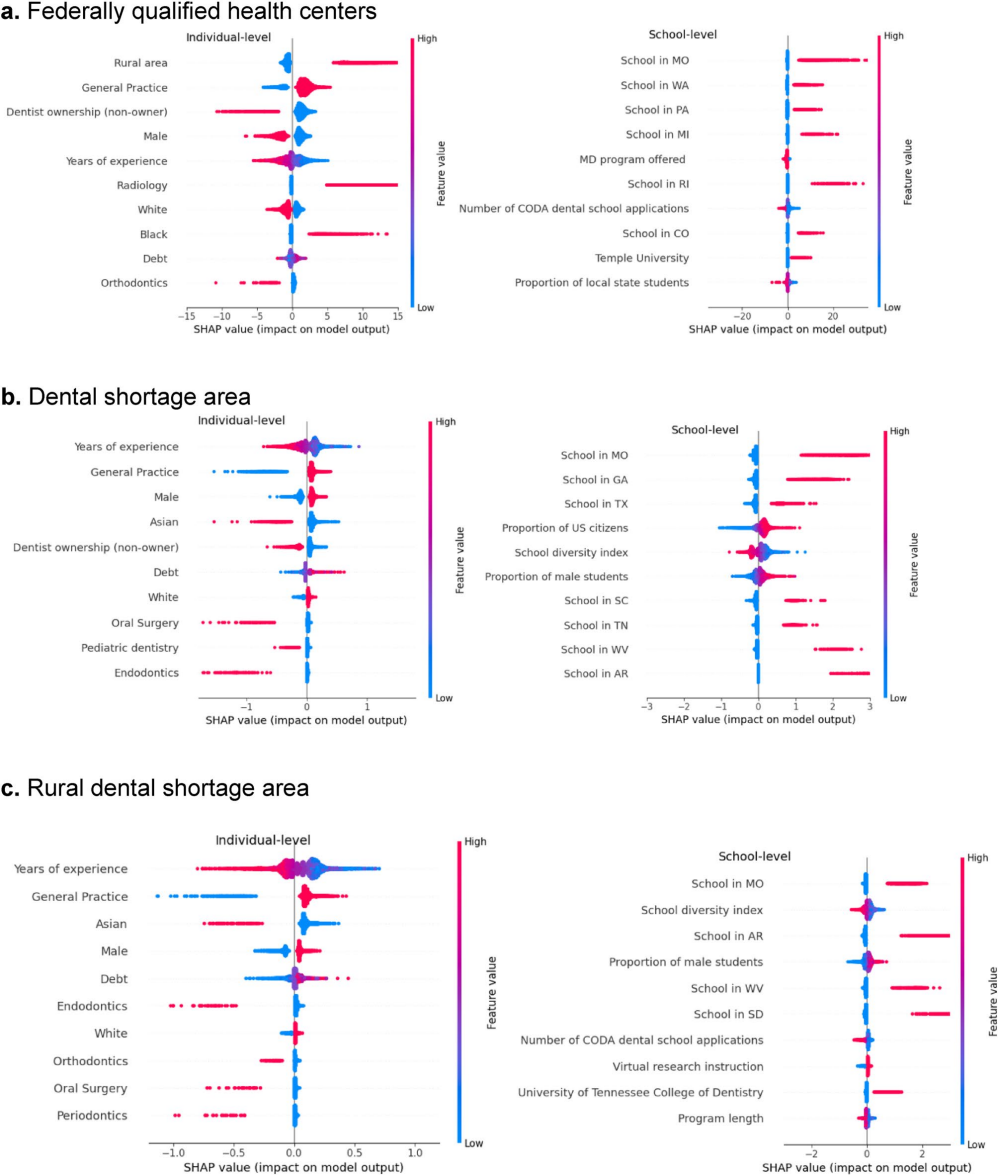
SHAP summary plots of the most important individual- and school-levelpredictors. *Note*. These SHAP summary plots display the contribution of key features to model predictions for individuals within the high-prevalence clusters. Features are categorized into individual-level and school-level groups and ranked by their average impact on the model’s output.

The SHAP analysis within high-prevalence clusters provided detailed information on key predictors (Fig. 3). For FQHCs, practicing in rural areas, general dentistry specialty, and non-ownership of practices were strong positive predictors. Other factors included female gender, fewer years of experience, Black race, and moderate debt. Graduating from dental schools in Missouri, Washington, Pennsylvania, and Michigan also increased the likelihood of FQHC practice, suggesting institutional impacts on workforce outcomes prediction. School-level factors, including offering an MD program, the number of CODA applications, and the proportion of local state students, also played a role.

For general dental shortage areas, influential predictors included fewer years of experience, general practice specialty, male gender, non-Asian ethnicity, non-ownership status, and high debt. Graduates from Missouri, Georgia and Texas dental schools were more likely to practice in shortage areas. Additional factors included higher US citizenship rates, lower school diversity index, and a greater proportion of male students.

For rural dental shortage areas, fewer years of experience, general practice specialty, non-Asian ethnicity, and male gender were the most significant individual-level predictors. Higher debt also had a significant impact, indicating financial considerations play an important role in decisions to practice rurally. Schools in Missouri, Arkansas, West Virginia and South Dakota were strong predictors. Other school-level predictors included diversity index, the proportion of male students, the number of CODA applications, and virtual research instruction.

The combined SHAP analysis (Supplemental Fig. 4) confirmed that individual-level factors were dominant across all models. General practice specialty, years of experience, rural background, and practice ownership consistently ranked as top predictors. Institutional factors, particularly dental schools in Missouri, West Virginia, and Arkansas, as well as broader characteristics like school diversity and virtual instruction, remained significant for rural and FQHC settings.

### SHAP heatmap and prediction interpretations

The SHAP heatmaps (Supplemental Fig. 5) provided a summary of significant predictors across sorted dentist instances. Consistently important predictors in FQHCs included rural practice, non-ownership of practice, and general practice specialty. For general dental shortage areas, the proportion of US citizens, general practice, and school diversity index had strong effects. Rural dental shortage areas predictors included general practice specialty, years of experience, and Asian ethnicity.

SHAP force plots (Supplemental Fig. 6) demonstrated how specific characteristics influenced individual predictions. Dentists with the highest predicted likelihood of practicing in FQHCs were recent graduates, practicing in rural areas, and attended dental schools in Missouri. In contrast, a lower likelihood was associated with greater years of experience and specialties such as pediatric dentistry. Similar interactions between demographic, financial, and educational factors shaped predictions for general and rural dental shortage areas.

### Debt and experience

**Fig. 4:**
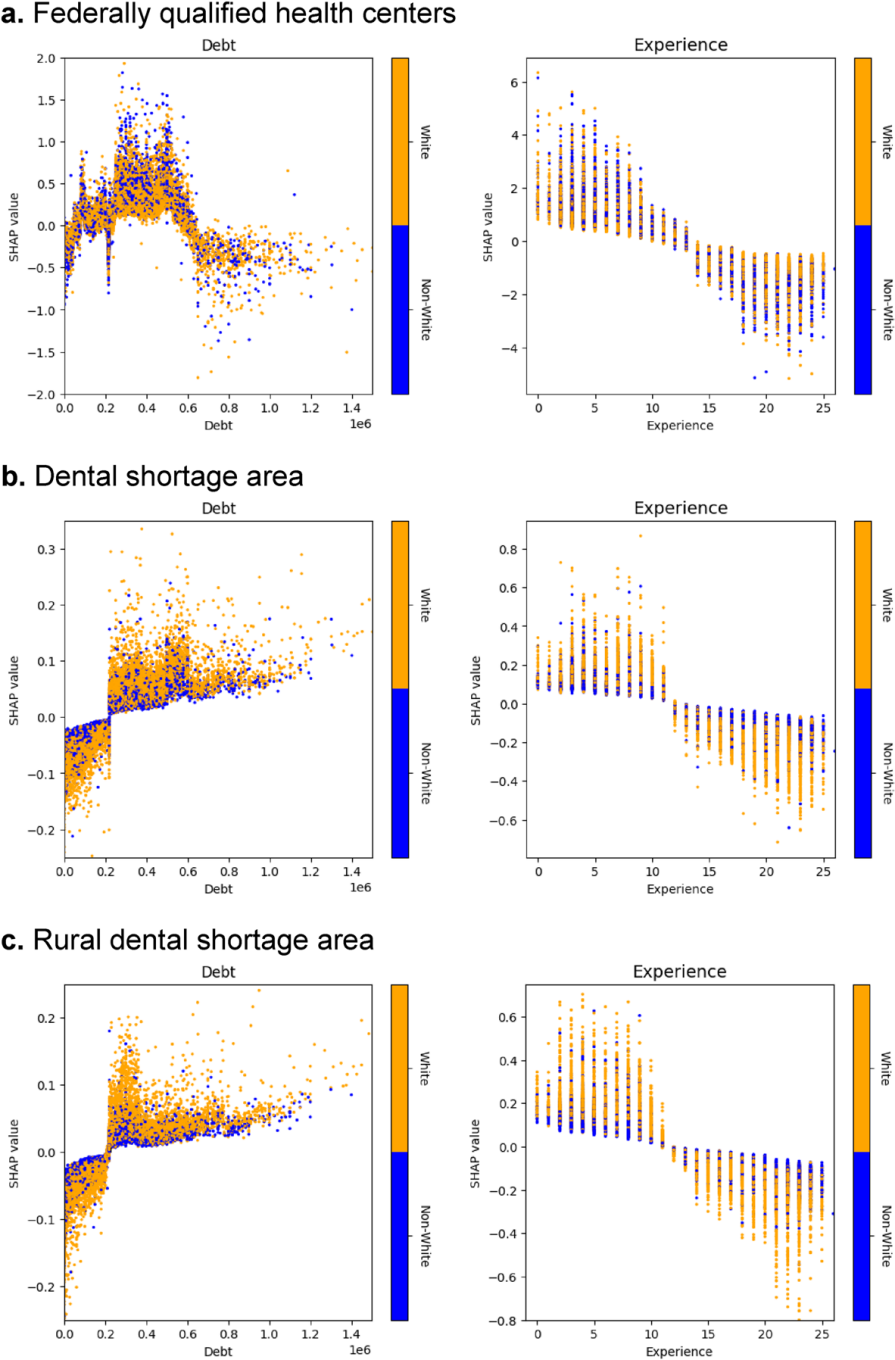
SHAP dependence plots of debt and years of experience for each outcome. *Note*. SHAP dependence plot shows the relationship between feature values and their corresponding SHAP values, which reflect each feature’s contribution to the model prediction. Each point represents an individual observation. The color bar indicates the values of an interacting feature—in this case, white ethnicity.

The relationship between debt and practice location differed by setting (Fig. 4). For FQHCs, moderate debt (~$200,000–$600,000) increased the likelihood of practicing, while very high debt (>$800,000) reduced this likelihood. In general dental shortage and rural shortage areas, higher debt was generally associated with a modest increase in the likelihood of practice, suggesting financial incentives might be less influential than in FQHC settings.

Years of experience had a strong inverse relationship with the probability of practicing in underserved areas. Early-career dentists (0–10 years) were more likely to work in all underserved settings. However, this likelihood declined significantly after 10–15 years of experience, mostly for FQHCs, highlighting potential challenges in long-term retention.

Color gradients in plots suggested racial disparities. White dentists showed greater sensitivity to financial incentives (debt levels), particularly in FQHCs. In contrast, non-White dentists showed more consistent practice decisions across career stages and debt levels, suggesting demographic differences in responsiveness to financial and professional incentives.

### Secondary analyses

Analyses restricted to individual-level predictors had lower predictive performance, with the best AUC of 0.69 for dental shortage and rural dental shortage areas, and 0.75 for FQHCs. Sensitivity, specificity, precision, and MCC were reduced, reflecting the importance of including school-level characteristics (Supplemental Table 2).

When analysis was limited to school-level predictors, performance remained robust, particularly for general dental shortage areas (best AUC = 0.80) and rural shortage areas (best AUC = 0.81). For FQHCs, the predictive performance was moderate (best AUC = 0.72). This result suggests the significant influence of institutional factors on dentist practice decisions in underserved areas.

## Discussion

Our findings highlight the complex interplay of factors shaping dentists’ decisions to practice in underserved areas, including FQHCs, general dental shortage areas, and rural dental shortage areas. Using machine learning models with strong predictive performance (AUCs ranging from 0.80 to 0.83), we identified key individual, institutional, and geographic determinants influencing dental workforce distribution. The SHAP-based model interpretability indicated that demographic characteristics (e.g., gender, race, years of experience), educational background (e.g., dental school diversity and institutional affiliation), and geographic context (e.g., state-level factors) all significantly affect practice location choices. These findings can support ongoing efforts and targeted policy interventions to address workforce maldistribution and improve access in underserved areas.

At the individual level, consistent predictors emerged across all underserved settings. General dentists were significantly more likely to work in underserved areas, which may reflect greater flexibility or fewer professional barriers compared to specialists. The likelihood of practicing in these areas declined with increased professional experience. This pattern supports prior findings that early-career dentists are more likely to begin their careers in underserved communities but transition to urban or private practice settings over time^24^. Additionally, non–practice owners were more likely to serve in underserved areas, possibly due to lower financial risk or fewer economic constraints compared to those who own their practices. Moreover, male dentists may be more willing to practice in shortage areas, possibly because life and family considerations play a greater role in practice location choices among female dentists. Gendered patterns in workforce participation and spousal employment opportunities may further shape these dynamics, particularly in rural regions where dual-career options are limited.^25^

Institution-level analyses showed that the impact of dental school background varied by type of the underserved practice. Graduating from dental schools in states like Missouri and West Virginia strongly predicted rural areas and general dental shortage area practice. These patterns likely reflect differences in state-level policies and training environments. States with stronger rural placement programs, community-based rotations, or loan repayment initiatives may cultivate a workforce more inclined to serve local needs. Additionally, higher school diversity indices were positively associated with practicing in FQHCs, suggesting that institutions commitment to diversity may help promote community-oriented values among their graduates. However, the negative association between school diversity and practice in general and rural shortage areas may reflect geographic and recruitment dynamics, dental schools with lower diversity are often located in less urbanized or resource-limited regions and tend to recruit students from nearby rural or disadvantaged areas. Graduates from such programs may have stronger local ties or a heightened sense of service to these communities.

Our findings expand upon previous literature, which primarily focused on individual-level factors such as personal background and economic motivations,^26,27^by emphasizing the role institutional characteristics play in influencing dentists’ practice choices. These findings align with initiatives such as East Carolina University School of Dental Medicine’s community-based training model and the Area Health Education Centers (AHEC) program, which aim to reduce disparities through targeted education strategies^24,28^. Addressing workforce disparities requires policies that integrate individual incentives with institutionally embedded curricula and outreach efforts.

The relationship between debt and practice decisions varied across settings. Moderate debt levels were positively associated with choosing FQHCs, likely due to targeted loan repayment incentives offered by these safety-net. In contrast, dentists with extremely high debt were less likely to pursue FQHC positions, pointing to concerns about financial sustainability. For general and rural dental shortage areas, higher debt generally increased likelihood of choosing these underserved settings, emphasizing the role of loan repayment programs in attracting dental professionals. This supports prior research linking educational debt to specialty choice and practice location^29^. Policymakers should continue to refine financial incentives, such as loan repayment and debt forgiveness programs, as strategic tools to recruit and retain dental professionals in underserved communities.

SHAP dependence analyses demonstrated important racial interactions. Among White dentists, higher debt was associated with more variations in underserved practice settings, suggesting differences in how economic viability or professional appeal are perceived across demographic groups. Additionally, the interaction between race and experience indicated that the influence of experience on practice location varied by racial and ethnic background. These patterns underscore the need for culturally informed outreach, recruitment, and retention strategies to reduce demographic disparities in the dental workforce. Our findings are consistent with previous research showing that minority dentists, especially those with personal connections to underserved communities, are more likely to serve these populations^30^. Addressing demographic disparities in the dental workforce requires targeted, culturally responsive policies.

### Limitations

This study has limitations. First, our dataset primarily included dentists who responded to the SDG. Self-reported data may be subject to recall bias, especially regarding debt or motivations for practicing in underserved areas. Second, our models are based on historical and current workforce data. Therefore, future shifts in healthcare policy or economic conditions might affect the applicability of our predictive models over time. Third, while SHAP analysis offers valuable insights into feature importance, it does not imply causality.

## Conclusion

This study demonstrates the potential of machine learning to guide dental workforce planning and inform policy interventions to improve equitable access to dental care. Key factors including demographic characteristics, educational background, institutional environment, and geographic influences significantly shape dentists’ practice-location choices. Addressing dental workforce disparities will require integrated strategies that combine individual incentives like loan repayment and mentorship with educational reforms, including admissions policies and community-based training. By identifying what drives dentists to serve underserved areas, policymakers can better tailor recruitment and retention efforts. Aligning incentives with dentists’ motivations and reinforcing educational pathways that emphasize community engagement can improve workforce distribution and promote oral health equity.

## Supplementary Information

Below is the link to the electronic supplementary material.

Supplementary Material 1

## Data Availability

The data supporting the findings of this study are restricted and not publicly available due to the data use agreement. However, for reasonable data requests, please contact the corresponding author.
