# Supplementary material for: Individual and institutional factors influencing dentists’ practice in underserved areas
Source: Sci Rep. 2026 Jan 5;16:2273. doi: 10.1038/s41598-025-32094-8 (PMC12816146; doi:10.1038/s41598-025-32094-8)
Supplement: Supplementary file 1 — Supplementary Material 1 [file 41598_2025_32094_MOESM1_ESM.pdf]

Supplement material

Individual and institutional factors influencing dentists' practice in underserved areas. Hawazin W. Elani, Ningsheng Zhao, Helena S. Schuch, Greg Saldutte, Elizabeth Mertz, Marko Vujicic.

**Supplemental Table 1**	List of variables included in the models
**Supplemental Table 2**	Model performance in predicting dentists' likelihood of practicing in underserved areas using individual-only and school-only predictors
**Supplemental Figure 1**	Datasets flow chart and linkage
**Supplemental Figure 2**	The structure of supervised clustering tree algorithm
**Supplemental Figure 3**	Inclusion curve of feature selection
**Supplemental Figure 4**	SHAP summary plot of most important predictors for high-prevalence group
**Supplemental Figure 5**	Heatmap of SHAP values based on sorted instances
**Supplemental Figure 6**	Examples of prediction interpretations for dentists with highest, lowest, and median prediction scores

Supplemental Table 1. List of variables included in the models

**Individual-level**
Age in years, gender, race, Hispanic, ownership, specialty, debt amount in $, experience in years, primary school type (US Dental; Foreign; Graduate), work in rural area, primary occupation, second occupation.
**School-level**
Dental school ID, dental school state, diversity index of dental school, total number of students enrolled, total proportion of male students, type of support (Public, Private Nonprofit, Private For-Profit, Private state-related), type of term (academic year; semester; quarter; trimester; other), type of degree (DMD, DDS), Program length in weeks, number of first-year students, number of CODA dental school applications, first-year enrollees of the CODA dental school applications, number of students of predental 2 years, number of students of predental 3 years, number of students of predental 4 years, number of bachelor students, number of master students, number of PhD students, number of students of other degree, number of students who received grants, total amount (dollars) in grants, virtual lecture offered, virtual simulation offered, virtual research offered, problem-based learning offered, case-based learning offered, service learning, live patients, number of patients, number of patients screened, DAT importance: academic, DAT importance: perceptual ability, DAT importance: science, DAT importance: quantitative, DAT importance: reading, DAT importance: biology, DAT importance: inorganic chemistry, DAT importance: organic chemistry, GPA importance: science, GPA importance: non-science, GPA importance: overall, interview importance, reference importance, mean DAT scores of first-year students in academic average, mean DAT scores of first-year students in perceptual ability, mean DAT scores of first-year students in science, GPA scores of first-year students in science, overall GPA scores of first-year students, the ratio of overall GPA /mean DAT (academic), the product of overall GPA and mean DAT (academic), PhD program offered, MD program offered, MPH program offered, MS program offered, other program offered, the proportion of local state students, proportion of US citizens, the proportion of Canadian citizens, the proportion of nonresident alien students, the proportion of students with unknown race, The proportion of students who applied for financial assistance, the acceptance rate of financial assistance application, total costs ($) of resident students, total costs ($) of nonresident students.

Supplemental Table 2. Model performance in predicting dentists' likelihood of practicing in underserved areas using individual-only and school-only predictors

Metrics	Federally qualified health centers	Dental shortage area	Rural dental shortage area
**Individual-level predictors**			
**Best Split**			
AUC (95% CI)	0.75 (0.73, 0.78)	0.69 (0.64, 0.74)	0.69 (0.62, 0.75)
Sensitivity	0.26	0.35	0.47
Specificity	0.96	0.82	0.74
Precision	0.23	0.02	0.01
Lift	5.02	2.01	1.78
MCC	0.21	0.05	0.04
**30 Splits**			
Average AUC	0.74 (0.73 - 0.74)	0.68 (0.67 – 0.69)	0.68 (0.67 – 0.69)
**School-level predictors**			
**Best Split**			
AUC (95% CI)	0.72 (0.70, 0.74)	0.80 (0.75, 0.84)	0.81 (0.76, 0.87)
Sensitivity	0.24	0.64	0.69
Specificity	0.95	0.80	0.79
Precision	0.20	0.04	0.02
Lift	4.19	3.27	3.03
MCC	0.17	0.13	0.10
**30 Splits**			
Average AUC	0.70 (0.69, 0.7)	0.76 (0.76, 0.77)	0.78 (0.77, 0.79)

Note. Model performance was evaluated using Monte Carlo cross-validation, with the dataset randomly split into 80% training and 20% testing across 30 iterations. "Best Split" refers to the single iteration with the highest performance, while "30 Splits" reflects the average performance across all iterations, offering a more robust and stable estimate of model accuracy.

Supplemental Figure 1. Datasets flow chart and linkage

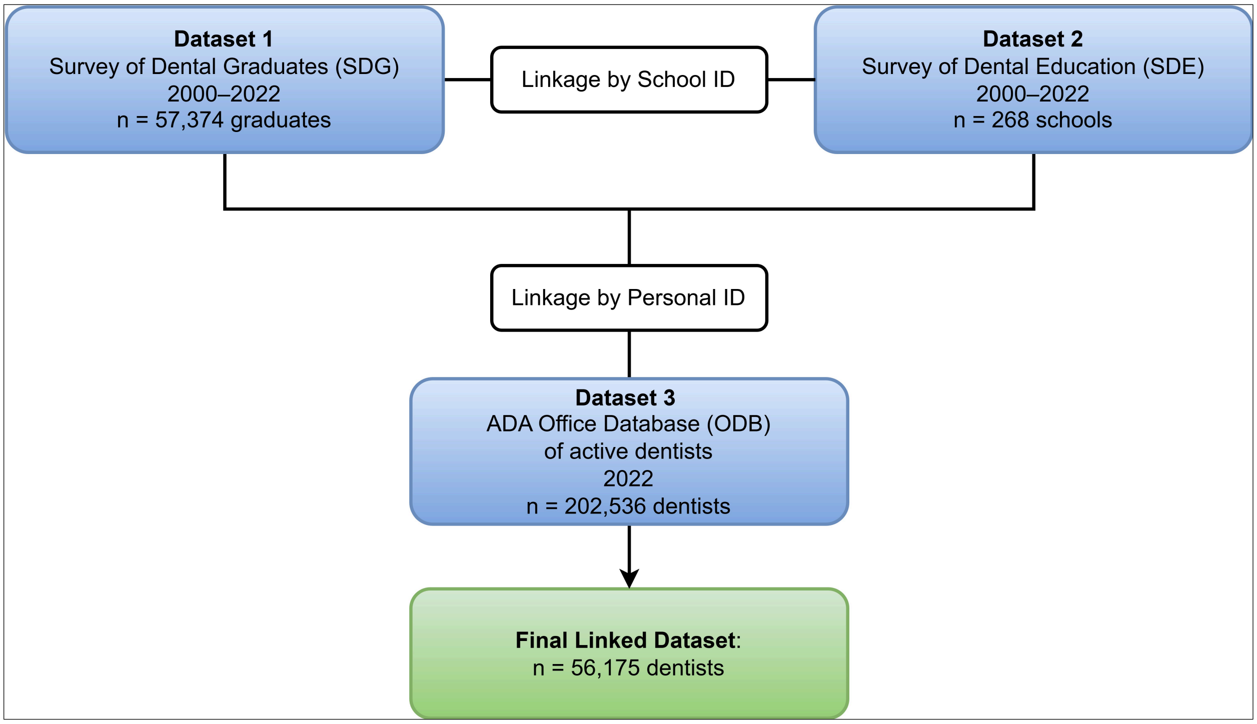

Supplemental Figure 2. The structure of supervised clustering tree algorithm

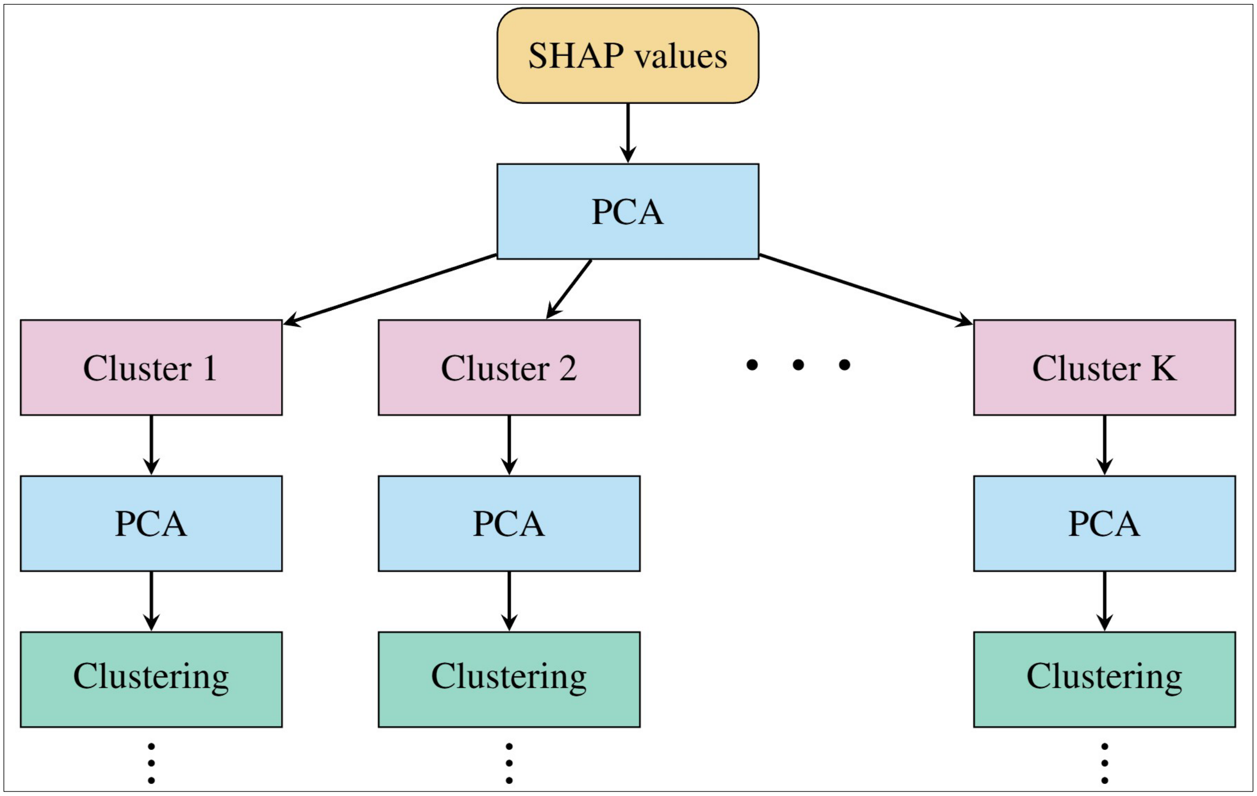

Note. The supervised clustering tree groups data based on SHAP values, incorporating model-driven insights rather than relying solely on raw data similarities. At each tree branch, Principal Component Analysis (PCA) is first applied to reduce the dimensionality of SHAP values within the parent cluster, followed by clustering to further partition the data. This hierarchical process enhances interpretability by progressively refining clusters based on feature importance derived from the model.

Supplemental Figure 3. Inclusion curve for feature selection

a. Federally qualified health centers

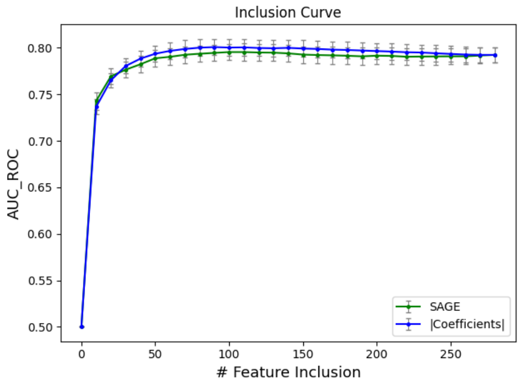

b. Dental shortage area

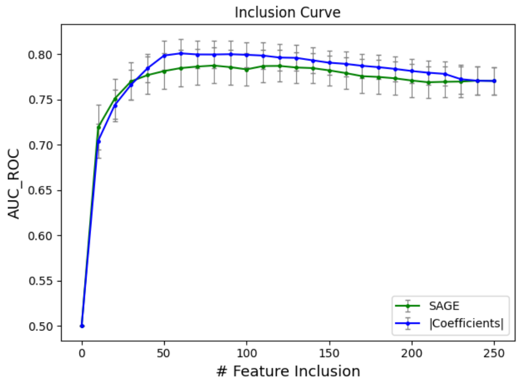

c. Rural dental shortage area

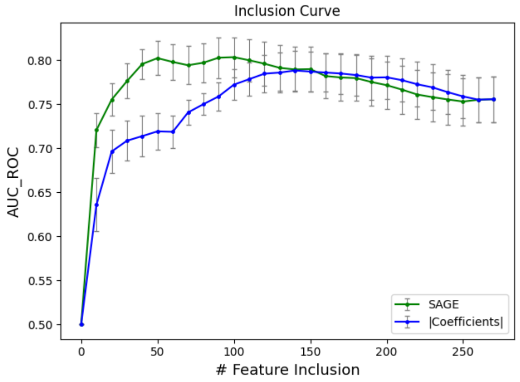

Note. Inclusion curves illustrate how model performance—measured by the average AUC-ROC across 30 random splits (with error bars indicating standard deviation)—changes as features are incrementally added based on their ranked importance. Feature importance was assessed using Shapley Additive Global Importance (SAGE) and the absolute values of model coefficients, respectively.

Supplemental Figure 4. SHAP summary plot of most important predictors for high-prevalence group

a. Federally qualified health centers

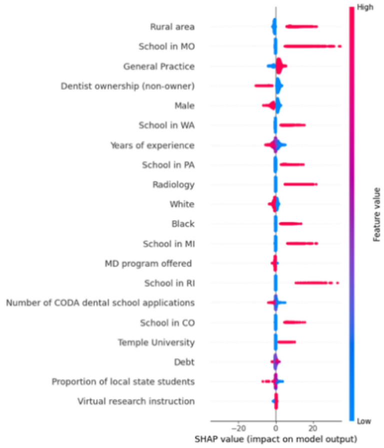

b. Dental shortage area

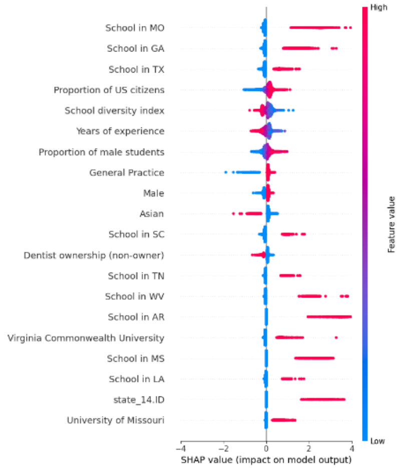

c. Rural dental shortage area

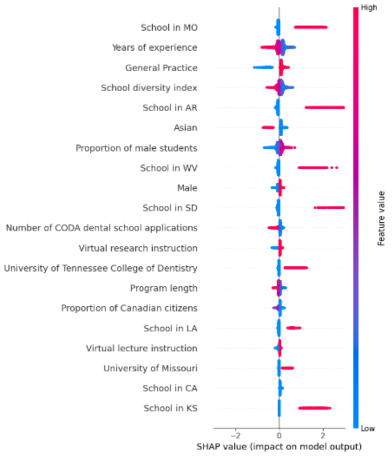

Supplemental Figure 5. Heatmap of SHAP values across sorted instances

a. Federally qualified health centers

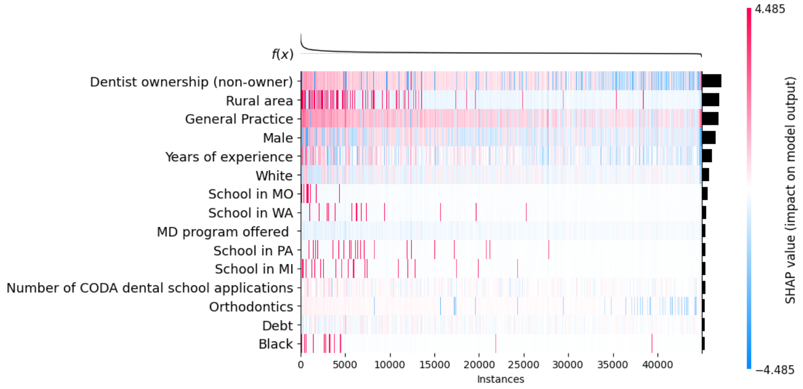

b. Dental shortage area

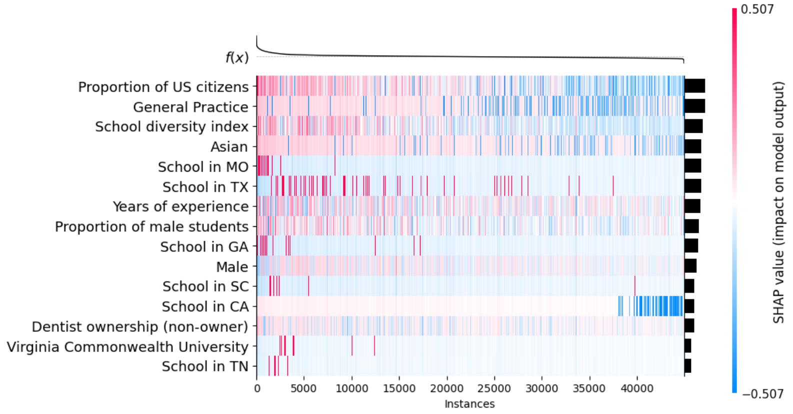

c. Rural dental shortage area

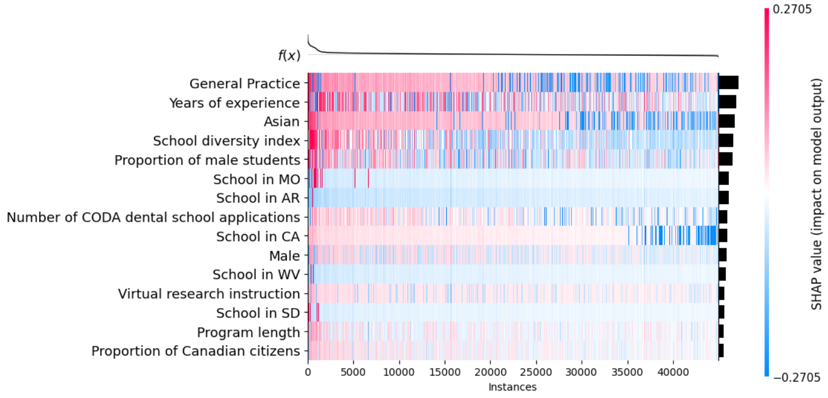

Note. This SHAP heatmap visualizes the contribution of each feature to the model's prediction for individual instances. Instances (x-axis) are sorted by their predicted scores, and features (y-axis) are ordered by the mean absolute SHAP value across all instances. Warmer colors (red) indicate stronger positive contributions to the prediction, while cooler colors (blue) reflect negative contributions.

Supplemental Figure 6. Examples of prediction interpretations for dentists with highest, lowest, and median prediction scores

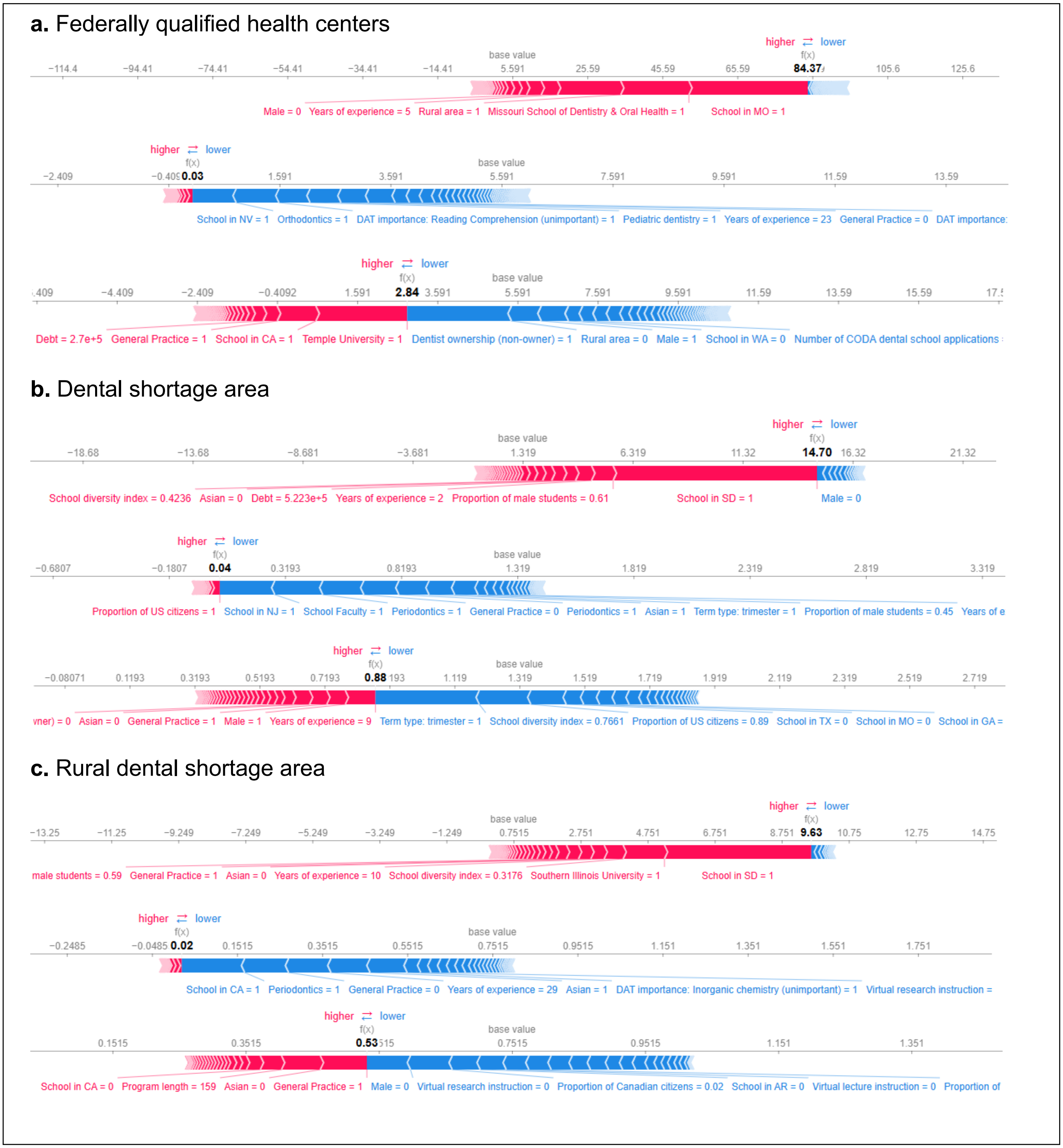

Note. SHAP force plots visualize the contribution of individual features to specific predictions. Features with positive SHAP values (in red) increase the predicted likelihood of practicing in underserved areas, while those with negative SHAP values (in blue) decrease it.
